# Data set on moral values and parental primary school choice: A study of Ado-Odo Ota, Local Government Area, Ogun State

**DOI:** 10.1016/j.dib.2021.107193

**Published:** 2021-06-02

**Authors:** Uju Abigail Ahaka, Oluyomi Ola-David, Uchechukwu Emena Okorie

**Affiliations:** a Department of Economics and Development Studies, Covenant University, Nigeria; b Fellow, Centre for Economic Policy & Development Research (CEPDeR), Covenant University, Nigeria

**Keywords:** Human capital, Education, Choice, School, Primary school, Parents

## Abstract

The Nigerian society has been infiltrated with poor public and private schools due to underfunding by government and intervention of private individuals who prioritize profit over qualitative education. The implication is on quality of human capital in the country which interconnect with social and economic development. The dataset examine how parents selects schools for their wards and investigated the influence of socio-economic status, school academic reputation, moral reputation of school and location of school. The data involve a multistage sampling technique and administered 250 questionnaires to parents. Structural Equation, Modelling was applied to test the influence of school academic performance, location, moral values and social economic status on parents’ choice of schools for their children and wards.

**Specification Table**

**Table:** 

Subject	Education
Specific subject area	Education has been widely seen to be a pivotal and an essntial part of an individual's life as it has embedded in it future benefits both for individuals and the collective benefits accruing to the society in which the individuals live in over time [1]. Moral values, parents’ location and school choice, academic performance, parents scoio-economic forms an integral components that can influence learning outcomes especially in the foundational level of education.
Type of data	Table Chart Figure
How data were acquired	Data were acquired using survey instrument and analysed with SEM in SPSS AMOS Version 22. The survey is uploaded to data in brief supplementary files.
Data format	Raw data is provided as an SPSS.SAV and uploaded as a supplementary document.
Parameters for data collection	Data were collected using structured research instrument administered with the aid of research assistants through direct self-completion questionnaire form. In order to ensure that the research instruments remained consistent, all the instruments were piloted so that corrections and modifications could be made. Consequently, the research design itself was ensuring that validity and reliability were addressed. Prior to field work involving data collection, permission was obtained from Covenant University relevant authorities in line with research ethics. No pressure was put on any respondent. Consent was sought from respondents. Confidentiality was assured in the whole process. The data collated were utilised strictly for academic and resaerch purposes as clearly stated in the consent form. All the instruments were piloted so that corrections and modifications were made to ensure that the research instruments remained consistent.
Description of data collection	The collection of data for the research work was through a well detailed and structured questionnaire written in English which contains structured questions in which the respondents filled. The questionnaires were filled-in anonymously as no personal questions regarding names, addresses and identity numbers were asked and consisted of a logical flow of questions which address matters relating to determinants of school choice. The nature of the research and the contents of the questionnaire were explained to the respondents. The researcher and field assistants through direct consultations with the respondents administered two hundred and fifty (250) questionnaire forms in the study area. A total of 215 participants were retained in the data set after cleaning and matching of the data.
Data source location	Department of Economics and Development Studies Covenant University, Ota, Ogun State South West Nigeria
Data accessibility	The data is hosted with the article.

## Value of the Data

• Data provide information on the socio-demographic characterisation, moral values, socioeconomic status, school location and performance that derives from theoretically-based estimation of parental primary school choice.
• Data provide empirical support for the determinants of parents and guardians choice of school at the basic level of education.
• Data is suitable for structural equation modelling, confirmatory and principal factor analysis with the application of robust goodness of fit model testing approach.
• Data were collected across divergent socio-economic characteristics which allows for test of significant difference on parental school choice among the cross-sectional participants using independent sample procedure for further insights
• Data can be further applied in categorical and ordinal analysis of other determinants of school choice by parents and guardians such as socio-economic status and location of schools.
• Data was collected across three categories of basic primary education thereby providing a platform for independent tests and measures changes across sample observations.

## 1 Data Description

The data are available as SPSS.SAV files. The data set consists of the constructs and indicators portrayed in Fig. 2. The descriptive statistic of the survey measures in the dataset is presented in Table 1. In the raw data the value “99” was a code used to capture indicators with the scale 1-5 in the missing value option using the SPSS-AMOS software application.

**Table 1:** Demographic information of the respondents in quantitative data.

Socio-demographic characteristics	Frequency (*n*=215)	Percentage %
Age		
18-30	45	21
31-40	72	33
41-50 51+	62 36	28 17
Sex		
Male	50	23.
Female	165	77
Occupation		
Formal employed	91	43
Self employed	122	58
Unemployed	2	6
Monthly income		
Less than #50,00	77	36
#51,000-#100,000	95	44
Above #200,000	16	7
Marital Status		
Never married	19	9
Married Divorced Widow	191 3 2	89 1.4 0.9
Education		
No formal education	2	0.9
Primary	18	8.4
Secondary	103	48
OND/TECH	23	11
HND/BSC Postgraduate	23 12	26 6

The data are contained as SPSS Statistics (*.sav) and csv files containing the raw data. The factors and item measures are listed with Fig. 2. The demographic and socio-economic identifications of the respondents are presented in Table 1. Table 2 shows the regression weights for the model constructs; choice of school (CS), school performance (SP), location of school (LN), religious moral values (MV) and employment status (ES) were further presented. The column heading “Label**”** in Table 2 shows the direction of influence among the variables, the estimated weights are represented as the ‘Estimate’**.** The standardized Error of Estimates (SE) of the respective study construct**,** the coefficient ratio (CR) measures the ratio of the estimate to SE while ‘P’ denotes the probability value of the estimates.

**Table 2:** Regression weights.

	Label		Estimate	S.E.	C.R.	P
CS	<—	SP	-.118	.052	-2.253	.024
CS	<—	LN	-.011	.060	-.191	.849
CS	<—	MV	.017	.025	.680	.496
CS	<—	SE	.067	.036	1.870	.061
Intercepts: (Group number 1 - Default model)						
	CS		1.929	.223	8.634	***

Note: *** indicates significance at 1% level of significance.

**Fig. 1:**
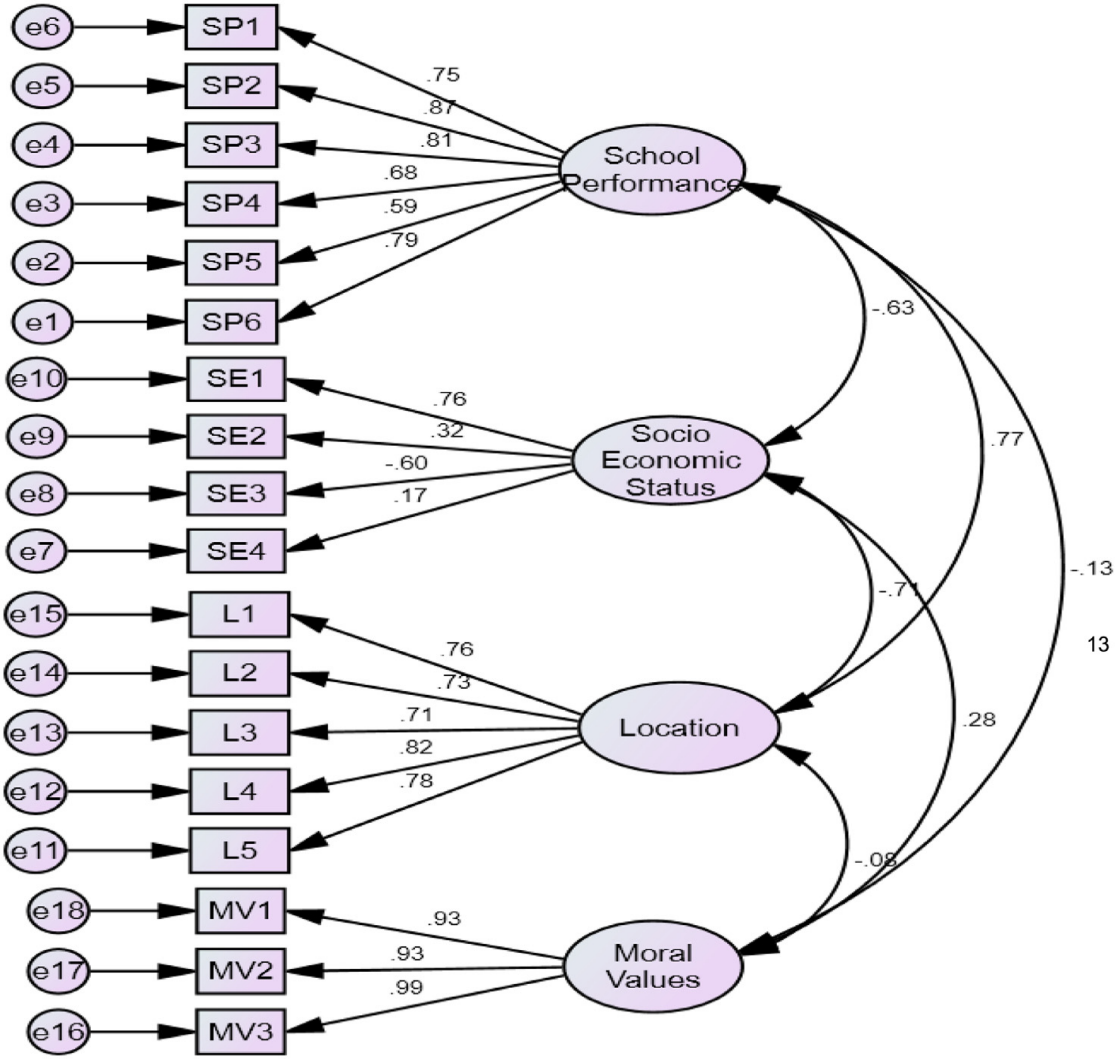
Coefficient of Confirmatory Factor Analysis. Where: SP1 = good exam result, SP2 = good impression, SP3 = curriculum, SP4 = specialized curricular activities, SP5 = special need (e.g. remedial classes), SP6 = good facilities, SE1 = Highest Educational attainment, SE2 = average monthly income, SE3 = Employment Status, SE4 = wealth influence the choice, L1 = Proximity to the school as a major determinant, L2 = Ease of transportation (public), L3 = the school is cheaper to reach, L4 = The location is safe (security), L5 = located in clean environment, MV1 = school's moral, discipline & religion, MV2 = religious belief influence my choice, MV3 = Religious values upheld by the school.

**Fig. 2:**
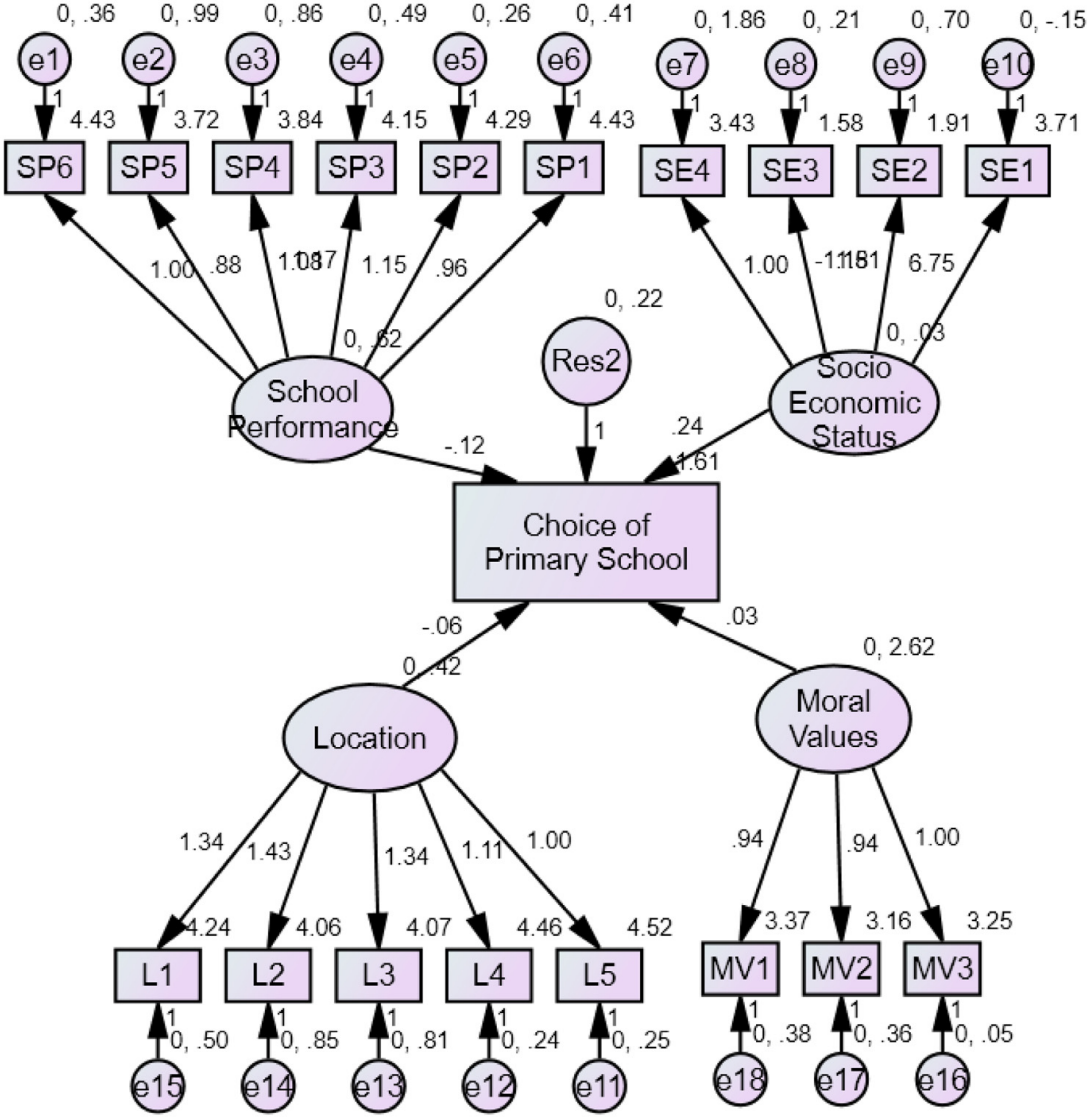
Structural equation model.

The measurement model for the confirmatory factor analysis (CFA) and validity of the indicators is presented in Fig. 1. In Fig. 1, school performance (SP) was measured with six indicators (SP1-SP6) and on their parent latent construct as one-factor model. Social economic status was measured with four indicators (SE1-SE4), location(L1-L5) was measured with five indicators and moral values (MV1-MV3) was measured with indicators and loaded with their latent constructs. The arrows connecting the factors with their items measured the extracted component of the inherent latent construct and direct causal relationship while the curved arrows measure the covariances among the factors. The items measuring the factors are enclosed in small rectangle s and the error terms in small circles while the bigger cycles are the latent factor domains. The path diagram of the variables in the structural model of the study is shown in Fig. 2.

The structural model in Fig. 2 indicates a structural path from the independent (school performance, economic status, location, moral values) to the dependent (choice of primary school). The independent factors are enclosed in circles while the dependent factor is enclosed in big rectangular box representing the global latent construct. The big arrows point to the causal relationship among items and its latent constructs factors and the smaller arrows represents the relationships between the items and the error terms located inside the small circles.

The ‘supplementary file’ contains a copy of the raw data in its original SPSS statistics (*.sav) format and also in .csv format attached with the manuscript.

## 2 Experimental Design, Materials and Methods

Survey research design was employed to acquire the perceptions of parents regarding the factors that determine their choices of primary school for their children. Furthermore, survey research design enables data to be collected that mirrors reality in contrast to secondary data which may not be reliable and have limited validity for research. The population concerned were the parents of Ado-Odo Ota Local Government Area whose children attend primary schools. The population was targeted at 50 parents from each of the five (5) selected areas in the Local Government summing up to 250 parents.

The data were retrieved from survey conducted in Ado-Odo Ota Local Government situated in Ogun State. Ado-Odo/Ota Local Government is one of the nineteen (19) local government areas in Ogun state with its headquarters at Ota. It came into being in 1989 following demands for more local government in the State. It is a grade A local government and the third largest in Ogun State, Ado-Odo/Ota Local Government [2]. It has the largest industrial area which generates the largest share of Internally Generated Revenue for the State. This becomes important to understudy the human capital level in the region with emphasis on school choice.

The local government selected which is Ado-Odo/Ota comprise of different communities [3]. Some include: Ado-Odo, Agbara, Igbesa, Iju-Ota, Itele, Kooko Ebiye, Owode, Sango, Ota, Attan, Iyana-Iyesi, Winners (Canaanland). Out of these, five communities were purposively selected, namely**:** Attan, Iyan-Iyesi, Winners, Ota, and Sango**.** In these communities, a community reconnaissance took place weeks before the commencement of the fieldwork. Permission was sought from heads of various schools within the local government areas selected. The community heads within the areas where the schools are located were also consulted, especially for permission, to avoid possible wrong perception about the data and for cooperation with our field assistants. However, only the schools where permission was granted were selected for the study. Recruited experienced research assistants administered the questionnaires. Participants’ recruitment followed research ethics and procedures. The selection of elements in the dataset entails multi Stage sampling technique. Cluster sampling and snow ball were employed given that Ado-Odo Ota local Government was clustered into Wards [4]. There are sixteen (16) wards in Ado Odo Ota. Five (5) out of these wards were selected using the simple random technique. They are Iju Attan, Iyan-Iyesi, Winners, Ota and Sango**.** Convenient sampling was used in administering the questionnaire to parents as they bring their wards to school in the morning. For parents who were illiterate, the questions were interpreted to them in the indigenous language (Yoruba) and their responses was recorded in to the questionnaires by the researcher.

Specifically, in terms of the respondents, three basic procedures were used in the selection of the respondents, namely intercept approach, respondent-accompanied system, and community random route-walk. In the first approach, parents were selected at random during the waiting time at the school-run hours. School runs is the period during which parents lead their children to school in the morning and afternoon at the school closing hours when they return to take their children back home. Interviews were only conducted among those who volunteered to be interviewed. Secondly, parents/guardians who fixed appointment for our research assistants were followed or accompanied to their residents or offices as the case may for the interview. This group of respondents gave their contact especially because of time exigency. Besides, respondents were also recruited through a random route-walk within the locations selected for the study. However, this was done using appropriate sampling intervals to selected houses and only one respondent was selected in each of the houses selected.

This study adopts the Cochran formula for sample size (calculation in smaller population),

$$n=\frac{n_{o}}{1+\left(n_{o}-1\right)/N}$$

where: n_o =_ Cochran's sample size

N = Population size

$$n=\frac{278}{1+\left(278-1\right)/1000}$$

$$n=\frac{278}{1+0.277}$$

n = 278/1.277 n = 217.7

The study however increased raised the sample size to 250. 250 questionnaires were distributed and 215 copies were retrieved.

After the data was collected, it was captured and loaded to the application software, Statistical Package for Social Sciences (SPSS) version 20. This is a software application programme that is used to enable the researcher to perform the tasks in a simple and easier manner. Frequency distribution and cross tabulation was produced. In addition, some data will be illustrated with the aid of graphs, pie charts and bar charts. In order to test the hypothesis, probability level of 0.5 and confidence level of 95% will be considered.

Based on the fact that the variable of interest in this study (school choice) cannot be measured perfectly or can be referred to as unobservable variables, the study employs Structural Equation Modelling (SEM) to test the hypotheses. The method is a multivariate statistical analysis which combines the factor analysis and multiple regression analysis to analyse structural relationships among variables. The analysis also estimates the multiple and interrelated dependence in a single analysis treating some of the variables as endogenous variables and exogenous variables. Also referred to as the casual modelling, the SEM test the proposed causal relationships. The goodness of fit used in this study is the Goodness of Fit Index (GFI).

The analysis covers the chi-square fit test in which the model will be considered a good fit if it has insignificant value, root means square error approximation (RMSEA), non-normed fit index (NNFI) and comparative fit index (CFI). The best model that will be selected will be based on the CFI and NNFI values exceeding 0.90 while the value of RMSEA below 0.05 are good for the model while values below 0.08 represent a model fit. Several reasons abound why structural equation modelling is beneficial, such as measurement errors that are explicitly taken into account, use of several indicators per construct simultaneously, and more so, confirmatory approach relationship including a multitude of hypotheses are tested simultaneously.

Definition of variables showing the abbreviations used in the Structural Equation Model

CS= Average for Parent Determinants of School Choice (Dependent Variable).

SP = Average of responses for School Academic Performance as a determinant of School

Choice (Independent Variable).

SE = Average of responses for Parents Socio-economic status as a determinant of School Choice (Independent Variable).

LN = Average of responses for Location of Parents as a determinant of School Choice

(Independent Variable).

MV= Average for Moral values as a determinant of School Choice (Independent Variable).

The validity of the socio-economic determinants of primary school measures was considered to measure facets of the four latent constructs to ascertain that the constructs are distinct from each other. The confirmatory factor analysis (CFA) was based on 18 items from the principal component analysis.

## Ethics Statement

Before going out in the field for data collection, permission was requested from Covenant University relevant authorities. No pressure was put on any respondent while the participants have the freedom to end participation any time without consequences. Consent was sought from respondents. Confidentiality was assured in the whole process. The findings of this study were strictly for academic purposes as clearly stated in the consent form.

## CRediT Author Statement

**Uju Abigail Ahaka:** Conceptualization, Investigation, Data curtion, Writing – original draft, Writing – review & editing, Resources, Supervision; **Oluyomi Ola-David:** Conceptualization, Supervision, Writing – review & editing, Validation; **Uchechukwu Emena Okorie:** Methodology, Software, Supervision, Writing – review & editing, Validation.

## Declaration of Competing Interest

The authors declare that they have no idea of any competing interest or personal influence which have, or could be perceived to have, affected the outcome presented in this article.

## Appendix Supplementary materials

Image, application 1

Image, application 2

Image, application 3
