# Supplementary material for: Data set on moral values and parental primary school choice: A study of Ado-Odo Ota, Local Government Area, Ogun State
Source: Data Brief. 2021 Jun 2;37:107193. doi: 10.1016/j.dib.2021.107193 (PMC8217690; doi:10.1016/j.dib.2021.107193)
Supplement: Supplementary file 3 [file mmc3.pdf]

CONSENT FORM

Dear parent/guardian,

I am a Postgraduate Student of Covenant University, Ota, conducting a survey on the Parental Preferences in the Choice of Primary School for their children/wards. The study is being conducted in order to provide relevant information for other parents, school owners/administrators and government towards the establishment of quality, and accessible primary school that can guarantee children/wards good academic performance.

Your participation in the study is voluntary, but we would be grateful if you could spare few minutes of your time to answer some relevant questions on this research. Any information supplied would be treated with utmost confidentiality and will have no legal implications.

Thank You.

Do you agree to participate? (1) Yes Signature (Optional): ___________ (2) No

If Yes, CONTINUE the interview. If No, (CLOSE INTERVIEW)

Appendix ii: SOCIO-ECONOMIC DETERMINANTS OF SCHOOL CHOICE: UNDERSTANDING HOW PARENTS CHOOSE PRIMARY SCHOOLS IN ADO-ODO OTA

SECTION A: Social Demographic Information (tick the appropriate answer)

1. Sex of respondent: Female [ ] Male [ ]

2. How old were you at your last birthday? (Indicate years) …………………………….

3. What is your current marital status?

Never married [ ] Married [ ] Divorced [ ] Widowed [ ] Separated [ ]

Cohabiting [ ]

4. What type of secondary school did you attend?

Public (Government) School [ ] Mission School [] Private School [ ]

5. What kind of primary school is your child/ward attending?

Public (Government) School [ ] Mission School [ ] Religious Private School [ ]

6. Who are you to your dependant (Relationship)?

Father [ ] Mother [ ] Guardian [ ]

7. What is the highest level of education have you obtained?

No formal education [ ] Primary School [ ] Secondary School [ ]

OND/Tech Schools [ ] 1st Degree (HND/BSc/BEng) [ ] 2nd Degree (MSc/MBA) [ ] PhD/DBA [ ]

8. Employment Status: Formal [ ] Informal/Self Employment [ ] Unemployed [ ]

9. What is your average monthly income?

Less than ₦50,000 [ ] ₦51,000 - ₦100,000 [ ] ₦ 101,000 - ₦ 200,000 [ ]

Above ₦200,000 [ ]

SECTION B: Below are statements regarding determinants of school choice, that is, factors that are anticipated could be influencing parents in their selection of primary schools for their children.

Please read each statement carefully and circle or tick one appropriate number that suits your opinion. Kindly make sure all statements are answered.

Use the following five-point scale of the agreement and disagreement with the statement. 1= Strongly Disagree, 2 = Disagree, 3 = Not Sure, 4 = Agree, 5 = Strongly Agree

S/N	DETERMNANTS OF SCHOOL CHOICE					
	School Academic performance influences school choice	1	2	3	4	5
10	I chose the primary school for my child because the school has good exam results/academic reputation					
11	I chose the primary school for my child because it has general good impression (it is attractive for various reasons e.g., facilities)					
12	I chose the school because of its curriculum & co-curricular activities such as clubs, sporting activities.					
13	I chose the primary school for my child because the school offers specialized curriculum e.g., music, dance, foreign language like French.					
14	I chose the primary school for my child because the school caters for special needs (e.g., remedial classes)					
15	I chose the primary school for my child because the school has small class sizes.					

16	I chose the primary school for my child because the school has good facilities for effective learning (e.g., science and computer laboratories).	
17	The type of primary school my child attends will affect the secondary school choice.	
	Parents' Socio-economic status	
18	My socio-economic status determined my choice of primary school for my child/dependant.	
19	My choice preference was a big factor in primary school choice for my child	
20	My spouse 's choice preference was a big factor in primary school choice for my child.	
21	My economic status (wealth) influenced the type of primary school I chose for my child.	
22	I consulted teachers of the chosen school to gather more information about it before choosing it.	
23	I collected information about the selected primary school through social networks/interactions with friends (e.g., at place of work or worship	

24	I chose the primary school for my child because that is where place was available.
25	I chose the primary school for my child because he/she (child) wanted to go there (i.e., following child 's choice).
26	I chose the primary school for my child because his/her friends went there.
27	I chose the primary school for my child because her/his sister or brother went there.
28	I chose the primary school for my child because I (or my spouse or other relatives) schooled there.
	Location (Residence) of Parents
29	Location (Residence) i.e. proximity to school is a determinant of school choice.
30	I chose the primary school for my child because it is near to my home.
31	I chose the primary school for my child where it is easy to get on public transport
32	I chose the primary school for my child where it is easy and cheaper to reach.

33	I chose the primary school for my child because its location is safe (There is security where the school is located).
34	I chose the primary school for my child because it is located in a clean environment.
35	I chose the primary school for my child because it is located in a conducive environment (e.g., the environment is quiet, there is no noise)
	School's moral values (e.g., discipline) and religious grounds
36	School 's moral values (e.g., discipline) and religious grounds influenced the choice of primary school for my child.
37	Religious values upheld by the school influenced my choice of school.
38	My religious beliefs influenced my primary school choice for my child
39	My choice of primary school was enforced by my devoutness to my religion
40	The primary school I chose constantly instils my values and belief system to my child/ward
41	The primary school is a branch of my religious institution.

42 The religious, moral and spiritual education provided for my child is the most important aspect of choosing my child/ward's primary school.

SECTION C - Suggestions

43. Do you think there are other factors that determine school choice at primary level other than what have been captured in the questionnaire? What are they?

....

....

....

....

....

....

....

44. Kindly indicate the name of school your child/ward is attending/attended:

………………………………………………………………………………………………………
